# *MYCL* is a target of a BET bromodomain inhibitor, JQ1, on growth suppression efficacy in small cell lung cancer cells

**DOI:** 10.18632/oncotarget.12671

**Published:** 2016-10-14

**Authors:** Fuyumi Kato, Francesco Paolo Fiorentino, Andreu Alibés, Manuel Perucho, Montse Sánchez-Céspedes, Takashi Kohno, Jun Yokota

**Affiliations:** ^1^ Genomics and Epigenomics of Cancer Prediction Program, Institute of Predictive and Personalized Medicine of Cancer (IMPPC), Campus Can Ruti, Badalona, Barcelona, Spain; ^2^ Genes and Cancer Group, Cancer Epigenetics and Biology Program (PEBC), Bellvitge Biomedical Research Institute (IDIBELL), Hospitalet de Llobregat, Barcelona, Spain; ^3^ Division of Genome Biology, National Cancer Center Research Institute, Tokyo, Japan

**Keywords:** small cell lung cancer, MYCL, JQ1, CDK6, cell cycle arrest

## Abstract

We aimed to elucidate the effect of JQ1, a BET inhibitor, on small cell lung cancers (SCLCs) with *MYCL* amplification and/or expression. Fourteen SCLC cell lines, including four with *MYCL* amplification, were examined for the effects of JQ1 on protein and gene expression by Western blot and mRNA microarray analyses. The sensitivity of SCLC cells to JQ1 was assessed by cell growth and apoptosis assays. *MYCL* was expressed in all the 14 cell lines, whereas *MYC/MYCN* expression was restricted mostly to cell lines with gene amplification. *ASCL1*, a transcription factor shown to play a role in SCLC, was also expressed in 11/14 cell lines. All SCLC cell lines were sensitive to JQ1 with GI_50_ values ≤1.23 μM, with six of them showing GI_50_ values <0.1 μM. Expression of *MYCL* as well as *MYCN*, *ASCL1* and other driver oncogenes including *CDK6* was reduced by JQ1 treatment, in particular in the cell lines with high expression of the respective genes; however, no association was observed between the sensitivity to JQ1 and the levels of *MYCL*, *MYCN* and *ASCL1* expression. In contrast, levels of *CDK6* expression and its reduction rates by JQ1 were associated with JQ1 sensitivity. Therefore, we concluded that *CDK6* is a novel target of JQ1 and predictive marker for JQ1 sensitivity in SCLC cells.

## INTRODUCTION

Small cell lung cancer (SCLC) is the most aggressive type of lung cancer with wide spread of metastases at the time of diagnosis in most cases [1]. Therefore, the development of novel systemic therapies is needed to improve patients' outcome in this deadly disease. Recent advances in target therapy of lung adenocarcinoma indicate that the gene products of activated oncogenes are promising targets for therapy of lung cancer patients [2-4]. One of three *MYC* family oncogenes, *MYC*, *MYCN* or *MYCL*, is amplified in approximately 20% of SCLCs, with the frequency of *MYCL* amplification the highest among the *MYC* family genes in clinical SCLC samples [5-9]. Amplification of the *MYCL* gene is usually accompanied by its overexpression in the corresponding tumors, while its expression is highly limited in adult tissues [10-12]. Therefore, *MYCL* seems an appropriate target of therapy in a subset of SCLC patients.

Bromodomain and Extra-Terminal domain (BET) proteins act as epigenetic signaling factors associated with acetylated histones and facilitate transcription of target genes. Recently, suppression of *MYC* and *MYCN* expression and activity by BET inhibition has been shown in several types of human cancers (reviewed in Shi J et al., 2014 and Fu LL et al., 2015) [13, 14]; however, its effects on *MYCL* have not yet been well studied. Therefore, we evaluated the efficacy of a BET bromodomain inhibitor, JQ1, on *MYCL* expression and the growth of SCLC cell lines with *MYCL* amplification. As a comparison for the effect of JQ1 in SCLC cells, two *MYC*-amplified cell lines, two *MYCN*-amplified cell lines and six cell lines without amplification of any *MYC* family genes were also used. MYC family proteins bind a DNA motif, E-box, as a heterodimer with a partner, MAX, to drive transcription of numerous target genes [15]. The *MAX* gene is inactivated by homozygous deletions in 6% of SCLCs mutually exclusively with *MYC* family gene amplification [16]. In 2 of the 6 cell lines without any *MYC* genes amplification, *MAX* was homozygously deleted.

We found that *MYCL* was expressed in all SCLC cell lines examined, including those without *MYCL* amplification. Furthermore, *MYCL* expression was considerably decreased by JQ1 treatment accompanied by growth reduction of the cells. Therefore, the *MYCL* gene is a target of a BET inhibitor, JQ1, and *MYCL* inhibition is a promising novel strategy for controlling the growth of a majority of SCLC cases. Moreover, *CDK6*, one of the driver oncogenes, was associated with the effectiveness of JQ1 on growth inhibition in SCLC cells.

## RESULTS

### Amplification/expression of *MYC* family genes/proteins

**Figure 1:**
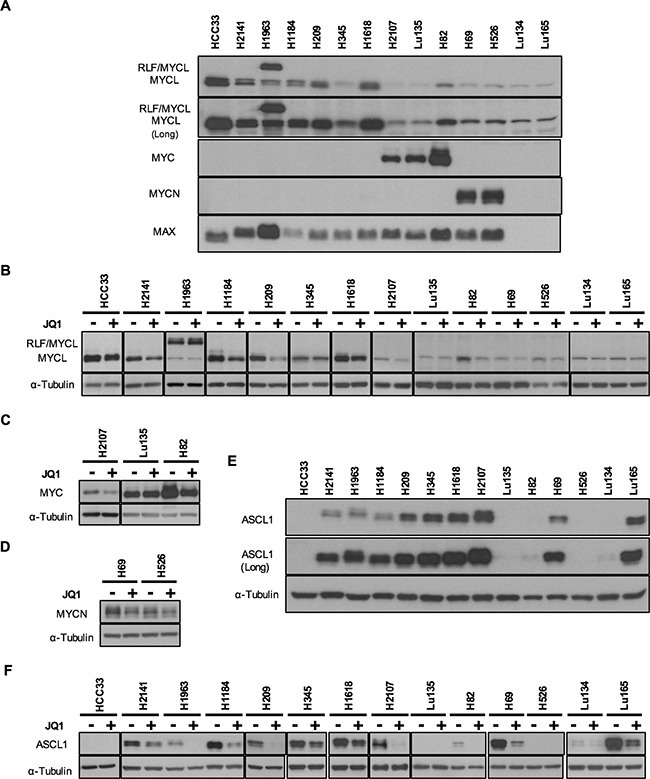
Expression of MYC family and ASCL1 proteins and their reduction by JQ1 in SCLC cell lines The results of Western blot analysis in 14 cell lines using antibodies for MYCL, MYC, MYCN, MAX and ASCL1 are shown. **A, E.** Expression of MYC family, MAX and ASCL1 proteins in 14 SCLC cell lines. To demonstrate the presence of MYCL protein in all the cell lines and ASCL1 protein in 11 of the 14 cell lines examined, the images of longer exposures (Long) are also shown. **B, C, D, F.** Reduction of MYCL, MYC, MYCN and ASCL1 expression by JQ1 treatment in SCLC cell lines. Cells were cultured for 24 hours with JQ1 (+) or without JQ1 (−), and whole cell lysates were analyzed using antibodies for MYCL (B), MYC (C), MYCN (D), ASCL1 (F) and α-Tubulin. Membranes were then incubated with a peroxidase-conjugated antibody. Enhanced chemiluminescence was performed according to manufacturer's instructions (Western Lighting Plus, Perkin Elmer).

**Figure 2:**
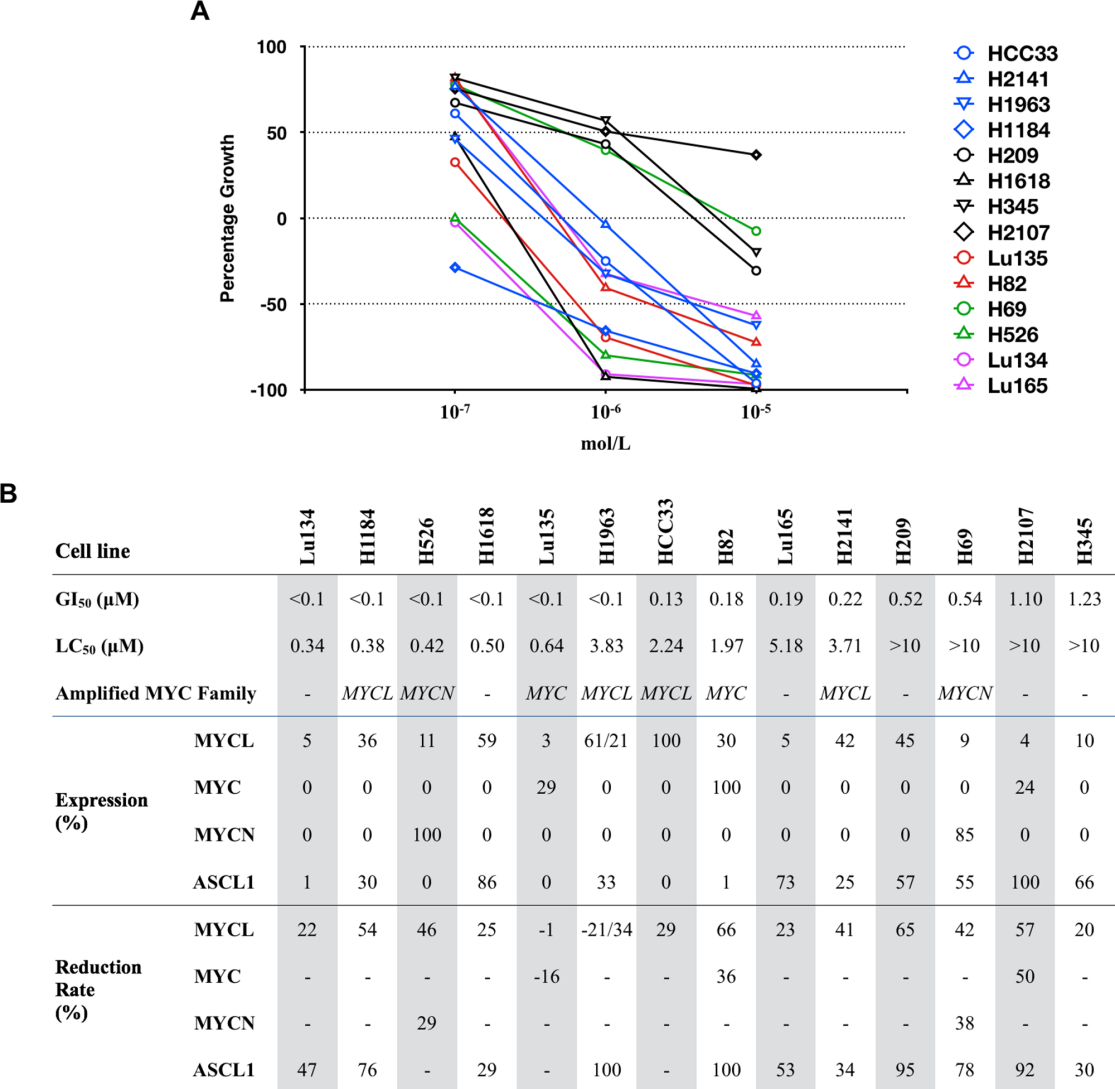
Effects of JQ1 on the growth and expression of MYCL, MYC, MYCN and ASCL1 proteins in SCLC cell lines **A.** Concentration response curves for JQ1 in 14 SCLC cell lines are shown. Blue, red and green symbols represent *MYCL*, *MYC* and *MYCN* amplified cell lines, respectively, purple symbols represent MAX deleted cell lines, and black symbols represent *MYC* family non-amplified cell lines. **B.** Cell lines are ordered according to the sensitivity to JQ1. GI_50_ and LC_50_ were calculated as described previously [18]. Amplified *MYC* family genes are indicated. Levels of MYCL, MYC, MYCN and ASCL1 protein expression and its reduction rates were calculated based on the amount of each protein normalized to α-tubulin shown in Figure 1 and Supplementary Figure S2. The relative protein levels in each cell line are expressed by the percentage of the level in the cell line with the highest expression among the 14 cell lines.

We previously reported the mutually exclusive amplification (copy numbers of 6 or more) of the three *MYC* family genes and homozygous inactivation of the *MAX* genes in the 14 SCLC cell lines used in this study (Figures 1 and 2) [8, 16]. *MYCL* was amplified in four cell lines, HCC33, H2141, H1963 and H1184. The *MYCL* gene was fused with the *RFL* gene in H1963 [8]. *MYC* was amplified in Lu135 and H82, and *MYCN* was amplified in H69 and H526, respectively. None of the three *MYC* family genes was amplified in the six other cell lines, H209, H345, H1618, H2107, Lu134 and Lu165. Among six *MYC* none-amplified cell lines, the *MAX* gene was homozygously deleted in Lu134 and Lu165 [16].

It has been shown that SCLC cells often express two *MYC* family genes together, even though only one of them is amplified in each tumor/cell line [17]. Therefore, we examined the expression of MYCL, MYC, MYCN and MAX proteins in these cell lines by Western blot analysis (Figure 1A). In contrast with MYC and MYCN, that only were expressed in three and two of the 14 cell lines MYCL was detected in all the 14 cell lines. This was evidenced by a longer time exposure of the membrane to the film. Band intensities varied considerably among the cell lines, and all four cell lines with *MYCL* amplification expressed abundant amounts of MYCL protein. In H1963, a larger size of MYCL protein was detected in addition to its normal. Since the *MYCL* gene was fused with the *RLF* gene and both *MYCL* and *RLF* were amplified in this cell line [8], the larger sized protein was likely to be an RLF/MYCL fusion protein. Among the 6 *MYCL* non-amplified cell lines, the levels of MYCL expression in H209 and H1618 were comparable to those in *MYCL* amplified cell lines. A relatively high level in H209 and a low level in H82 of MYCL expression were previously reported [17], consistent with the present result.

MYC protein was detected in two *MYC* amplified cell lines, Lu135 and H82, and MYCN protein was detected in two *MYCN* amplified cell lines, H69 and H526. Among 10 *MYC/MYCN* non-amplified cell lines, MYC was expressed only in the H2107 cell line, and neither MYC nor MYCN were detected in the remaining nine cell lines. MAX protein was absent in two cell lines with *MAX* deletions, Lu134 and Lu165, and these cell lines expressed only small amounts of MYCL, but not MYC and MYCN. Therefore, both MYC and MYCL proteins were expressed in the H2107, Lu135 and H82 cell lines, and both MYCN and MYCL proteins were expressed in the H69 and H526 cell lines. However, none of the cell lines expressed both MYC and MYCN proteins. In the H82 *MYC* amplified cell line, a relatively high level of MYCL protein was detected in addition to an extremely high level of MYC protein.

### Growth inhibition induced by JQ1

To evaluate the sensitivity of SCLC cells to JQ1 for their growth inhibition, the cells were cultured with JQ1 at concentrations of 10.0, 1.0 or 0.1 μM, and the GI_50_ values as well as LC_50_ values were calculated in each cell line. GI_50_ is the concentration of JQ1 that causes 50% growth inhibition, relative to the control without JQ1, while LC_50_ is the concentration of JQ1 that cause lethality in 50% of the cells at the time of plating [18]. Growth reduction was observed in a dose dependent manner in all the 14 cell lines examined (Supplementary Figure S1). Therefore, dose-response graphs for JQ1 in these cell lines were drawn (Figure 2A), and GI_50_ and LC_50_ were calculated according to the methods described previously (Figure 2B) [18]. The GI_50_ values ranged from <0.1 μM to 1.23 μM. None of them were highly resistant to JQ1 (GI_50_ >10 μM). In particular, six of the 14 cell lines were highly sensitive to JQ1 (GI_50_ <0.1 μM). With a high concentration of JQ1 of up to 10 μM, the cell number after several days of JQ1 treatment was less than that at the time of plating in all cell lines except H2107, indicating that a significant fraction of the cells died by JQ1 treatment in these cell lines. These results imply that a high concentration of JQ1 in culture *in vitro* is lethal to SCLC cells.

### Cell cycle arrest and apoptosis induced by JQ1

**Figure 3:**
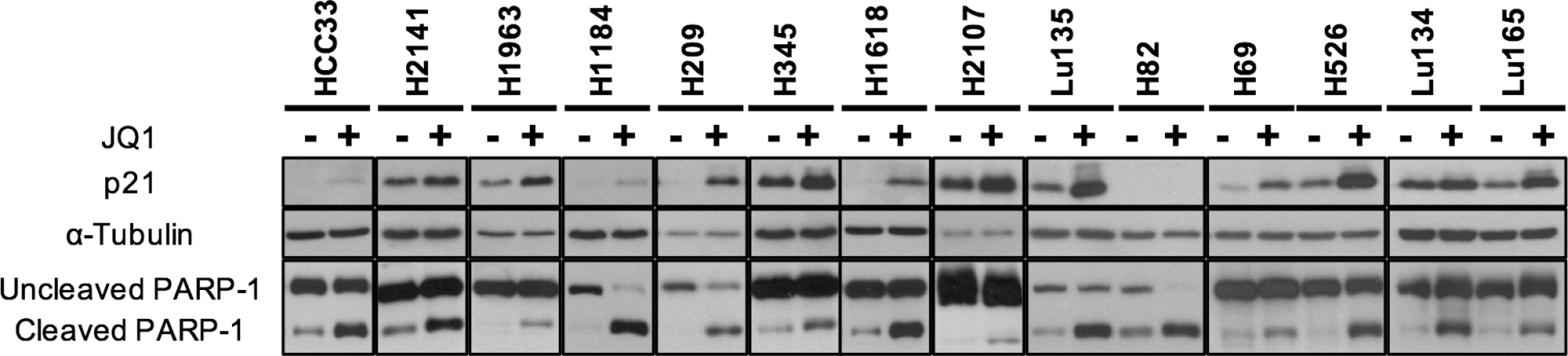
p21 expression and PARP-1 cleavage enhanced by JQ1 treatment in SCLC cell lines The results of Western blot analysis in 14 cell lines with (+) and without (−) JQ1 using antibodies for p21, α-Tubulin and PARP-1 are shown. Experimental details as in Figure 1.

Cell cycle arrest and/or apoptosis have been shown to occur in several types of cancer cells by JQ1 treatment [13, 14]. p21 induction and PARP-1 cleavage are well established indicators of G1 arrest and apoptosis, respectively, induced by JQ1 treatment. To elucidate whether growth reduction caused by JQ1 in SCLC cells was a consequence of the induction of cell cycle arrest and/or apoptosis, Western blot analysis was performed using antibodies for p21 and PARP-1. The amounts of p21 as well as the percentages of cleaved PARP-1 were assessed after 24 hours of 10 μM JQ1 treatment in all the cell lines. As shown in Figure 3, the amounts of p21 increased in all cell lines except H82. Bands for p21 were undetectable or very faint in HCC33, H1184, H209, H1618 and H82 under standard culture conditions. However, bands for p21 became detectable in all cell lines except H82 after JQ1 treatment. In the remaining nine cell lines, p21 was detected even without JQ1 treatment under standard culture conditions, and its amounts were evidently increased after JQ1 treatment. The percentages of cleaved PARP-1 fragments were also increased by JQ1 in all the cell lines. These results support the induction of G1 arrest and eventually apoptosis by JQ1 treatment in these cell lines, consistently with the results of the growth assay. Therefore, we concluded that JQ1 has an activity to induce cell cycle arrest and apoptosis in SCLC cells.

### Reduction in the amounts of MYC family proteins by JQ1

To evaluate whether JQ1 has an effect to inhibit MYC family protein expression in SCLC cells, we performed Western blot analysis of JQ1 treated and non-treated cells with antibodies for MYCL, MYC and MYCN (Figure 1B-1D). The cells were collected after 24 hours of culture with or without 10 μM of JQ1. The reduction rates for each of MYC family proteins were normalized to the levels of α-Tubulin. MYCL band intensities were reduced by 20% to 66% by JQ1 treatment in all the cell lines except Lu135, suggesting that the *MYCL* gene is a target of JQ1 for transcriptional silencing in the majority of SCLC cell lines (Figure 2B). The H1963 cell line expressed both the RLF/MYCL fusion protein and the wild-type MYCL protein. Whereas the amount of wild-type MYCL protein was reduced by JQ1 (34%) as in other cell lines, the amount of RLF/MYCL fusion protein was not reduced (Figure 1B and 2B), suggesting that the fused *RLF/MYCL* gene is not a target of transcriptional silencing by JQ1. The amounts of MYC protein were slightly reduced in two of the three cell lines with MYC expression, but not in Lu135. The amounts of MYCN protein were reduced in both H69 and H526 cell lines with *MYCN* amplified and overexpressed.

Accordingly, we concluded that, with the exception of MYCL and MYC in Lu135 and RLF/MYCL in H1963, the expression of all three MYC family proteins was reduced by JQ1 treatment in all SCLC cell lines examined.

### ASCL1 expression and its reduction by JQ1

Achaete-scute homolog 1 (ASCL1) is a transcription factor necessary for the development of neuroendrocrine cells and the growth of SCLC cells [19]. It was recently reported that ASCL1 is a critical target of JQ1 for transcriptional silencing in SCLC cell lines [20]. Therefore, ASCL1 expression was also examined by Western blot analysis in our panel of SCLC cell lines (Figure 1E). ASLC1 was expressed in 11 of the 14 cell lines (79%), with its levels varying among the cell lines. Faint bands for ASCL1 were detected after a long time exposure in H82 and Lu134 cells and no bands were detected in HCC33, Lu135 and H526 cells. Expression profiles of ASCL1 in these 14 cell lines were very similar to those of SCLC tumors, showing that the majority (77%) of the tumors expressed high levels of ASCL1 [7]. Protein levels of ASCL1 were apparently not associated with those of any MYC family member in these cell lines. Therefore, the effect of JQ1 on ASCL1 expression was further examined by Western blot analysis (Figure 1F). The amounts of ASLC1 protein were reduced in all the 11 cell lines that expressed ASCL1 under standard culture conditions (Figure 2B).

### Association of JQ1 sensitivity with MYC family and ASCL1 protein expression

In order to predict the sensitivity of SCLC cells to JQ1, it is important to clarify whether *MYC* family gene amplification/expression and/or ASCL1 expression are associated with the growth inhibition effect of JQ1 to SCLC cell lines. Since two of the four *MYCL* amplified cell lines, one of the two *MYC* amplified cell lines, one of the two *MYCN* amplified cell lines, and two of the six MYC non-amplified cell lines were highly sensitive to JQ1 (GI_50_ <0.1 μM) (Figure 2), sensitivities to the drug did not seem to be associated with amplification of any *MYC* family genes. Furthermore, to assess the association of JQ1 sensitivity with the levels of MYCL, MYC, MYCN and ASCL1 proteins, the relative expression of these proteins to the cell line with the highest expression among the 14 cell lines was calculated (Figure 2B) and compared between the 6 cell lines with high sensitivity to JQ1 (GI_50_ <0.1 μM) and the 8 cell lines with low sensitivity to JQ1 (GI_50_ ≥0.1 μM). None of these proteins showed an association between their levels of expression and the sensitivities to JQ1 (Supplementary Figure S3).

### Association of JQ1 sensitivity with *CDK6* mRNA expression and its reduction by JQ1

JQ1 is known to induce growth suppression by transcriptional inhibition of numerous genes, including *MYC*, *MYCN* and *ASCL1*, in human cancer cells [13, 14, 20-25]. To clarify whether *MYCL* as well as *MYC*, *MYCN* and *ASCL1* were transcriptionally suppressed by JQ1 in SCLC cells, we further performed mRNA microarray analysis in 12 of the 14 SCLC cell lines, which were collected after 24 hours of culture with and without 1.0 μM of JQ1. As summarized in Supplementary Table S1, *MYCL* as well as *MYCN* and *ASCL1* were transcriptionally suppressed by JQ1, in particular, in cell lines with high expression levels (>1,000 in control cells). In contrast, transcriptional suppression was not detected in the *MYC* gene, as reported previously in SCLC cell lines [20], even in Lu135 and H82 carrying *MYC* amplification and overexpression. The results of mRNA microarray analysis were overall similar to those of Western blot analysis in most of cell lines, with some variations probably because of culture and experimental conditions. The result indicates that *MYCL* is also a target of JQ1 for transcriptional silencing in SCLC cells. However, even though *MYCL* was overexpressed in H1963, no reduction was observed by JQ1, consistent with the result of Western blot analysis, supporting that the rearranged/fused *MYCL* gene in this cell line is not a target of JQ1, likely because of the use of the *RLF* promoter for transcription.

*FOSL1*, *BCL2*, *CDK6*, *IL7R*, *RUNX2* and *CDC25B* have been shown to be targets of JQ1 for transcriptional silencing in other types of cancers [23, 26-31]. Therefore, we also examined the effect of JQ1 on transcriptional levels of these genes (Supplementary Table S1). The expression of *FOSL1*, *IL7R* and *RUNX2* was very low and not markedly changed by JQ1. However, the expression of *BCL2*, *CDK6* and *CDC25B* was high (>1,000) in several cell lines, and their levels were decreased by JQ1 treatment in most cell lines, in particular those with high levels of expression. Namely, expression of *BCL2* was markedly reduced by JQ1 in H345 and H2107, while expression of *CDK6* was reduced by JQ1 in H1618, Lu135, H82, H526 and Lu134. Expression of *CDC25B* was reduced by JQ1 in 11 of the 12 cell lines.

Therefore, we further investigated the association between the reduction rate of expression and the GI_50_ values at 1.0 μM of JQ1. Only *CDK6* showed a significant association (p=0.003 by Pearson correlation test), while none of *MYCL*, *MYCN* or *ASCL1* showed such an association. We also investigated the association between the expression levels in non-treated cells and the GI_50_ values. *BCL2* (p=0.011) and *CDK6* (p=0.002) were predictive markers for the effectiveness of JQ1 on growth inhibition, although the association with *BCL2* expression was much weaker than that with *CDK6* expression. Therefore, we conclude that *CDK6* plays a common role for the growth inhibition by JQ1 in most SCLC cells. Thus, the sensitivities to JQ1 for growth inhibition could be in part defined by the levels of *CDK6* expression and its reduction rates in SCLC cells.

**Figure 4:**
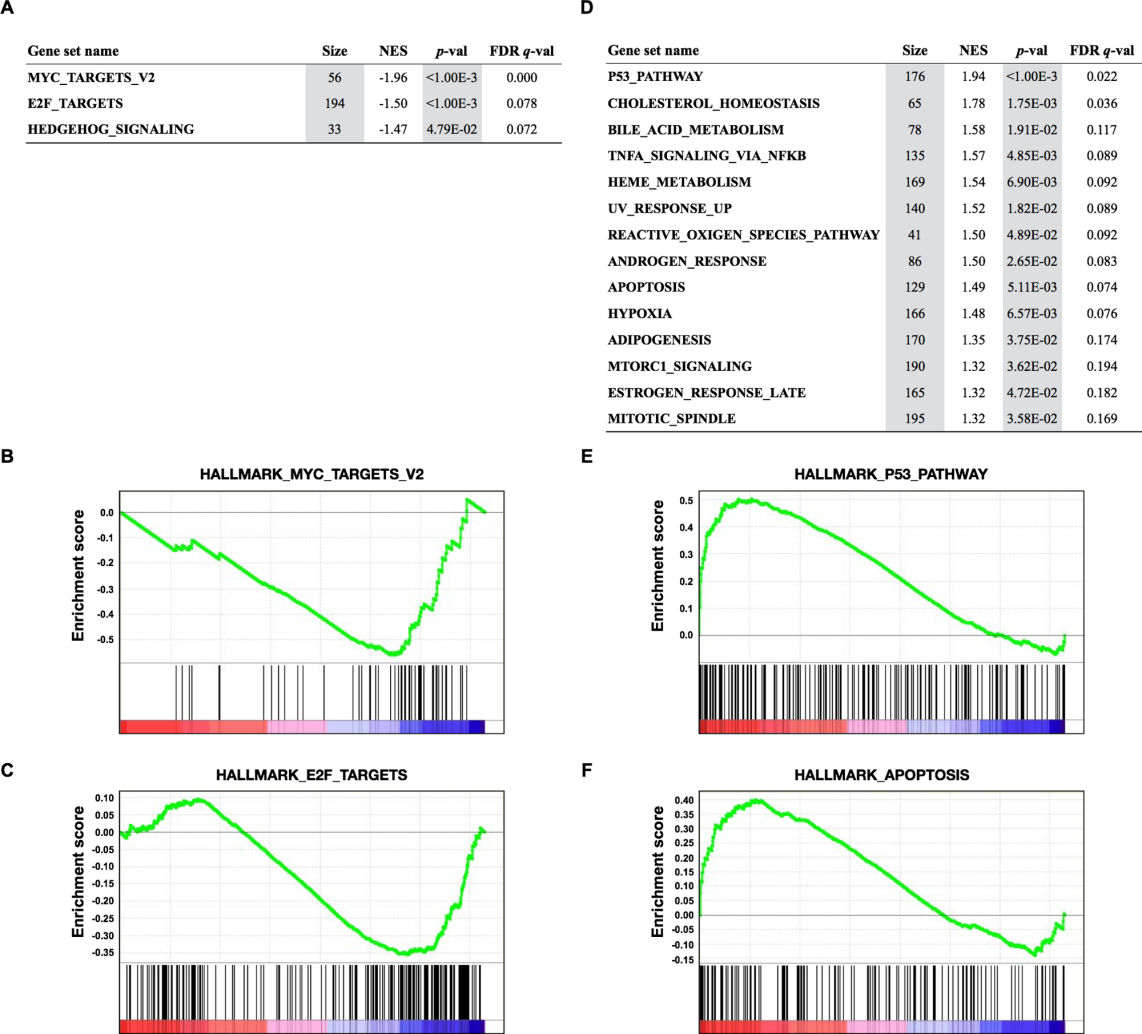
Gene Set Enrichment Analysis (GSEA) of transcriptional profiles in 12 SCLC cell lines treated with JQ1 **A, D.** Gene sets significantly enriched (*p*<0.05) among genes downregulated (A) and upregulated (D) by JQ1 in 12 SCLC cell lines with *p*-value, FDR *q*-value, the number of genes (Size), and the normalized enrichment score (NES). **B, C, E, F.** GSEA plots showing downregulation of MYC (B) and E2F targets (C), and upregulation of P53 (E) and apoptosis (F) pathways.

To examine whether JQ1 specifically abrogates MYC-dependent transcription and which pathways are affected by JQ1, we further performed gene set enrichment analysis (GSEA) to identify functional gene sets with significant downregulation or upregulation by JQ1 treatment (Figure 4) (http://software.broadinstitute.org/gsea/msigdb/collections.jsp). GSEA revealed a significant downregulation of 3 of the 50 hallmark gene sets. HALLMARK_MYC_TARGETS_V2 was the most significantly downregulated pathway, supporting the conclusion that *MYC* family genes were transcriptionally inhibited by JQ1 in SCLC cells. The second most significantly downregulated pathway was HALLMARK_E2F_TARGETS. Gene sets significantly upregulated by JQ1 included HALLMARK_P53_PATHWAY and HALLMARK_APOPTOSIS. These results support the occurrence of G1 arrest and apoptosis in SCLC cells by JQ1 treatment.

## DISCUSSION

In this study, we first determined the expression of the three MYC family proteins in 14 SCLC cell lines by Western blots. We found that MYCL protein was expressed in all the cell lines indicating that MYCL is expressed widely in SCLC cells, even though the *MYCL* gene may not be amplified. In contrast, MYC and MYCN were expressed mostly in cell lines with amplification of the respective genes. Other than SCLC, amplification and/or expression of *MYCL* have been detected only in ovarian and gastric cancer [32, 33], although its biological significance is still unknown. We and others have demonstrated the growth inhibition of SCLC cells *in vitro* by blocking MYCL [6, 34]. Thus, this and previous studies point to *MYCL* as a promising target of therapy specifically in SCLC patients.

Then, we assessed the sensitivity of these SCLC cell lines to a bromodomain inhibitor, JQ1, since the effects of JQ1 on the reduction of *MYC* and *MYCN* expression associated with growth inhibition have been reported in several other types of cancers [13, 14, 21-25]. Consistent with the results of recent studies by others [20, 35], growth reduction of SCLC cells was highly sensitive to JQ1, indicating the potential utility of bromodomain inhibitors as therapeutic drugs for SCLC patients. Therefore, we further examined the effect of JQ1 on the expression of *MYCL*, as well as *MYC* and *MYCN*, in these cell lines. Since the inhibitory effect of JQ1 on the expression of *ASCL1* in SCLC cells was recently reported [20], we included *ASCL1* in our analysis. Expression of *MYCL* as well as *MYCN* and *ASCL1*, but not *MYC*, was markedly reduced by JQ1 at both protein and mRNA levels. Therefore, it is likely that SCLC cells are generally highly sensitive to JQ1, which can be implemented by the combined expression of multiple genes, including *MYCL*, *MYCN* and *ASCL1*.

The results indicate that in SCLC cells, multiple genes involved in cell growth are transcriptionally silenced by JQ1, and *MYCL* is one of them. However, the relative sensitivities to JQ1 were not associated with the expression of any of these genes in our panel of 14 SCLC cell lines. On the other hand, we identified *CDK6* and *BCL2* as JQ1 targets for transcriptional silencing in SCLC cells, as in leukemia [23, 36], lymphoma [37] and neuroblastoma [30]. In particular, the levels as well as reduction rates of *CDK6* expression showed the strongest association with JQ1 sensitivity among the genes examined. The detection of *CDK6* as a novel target for the transcriptional inhibitory role of JQ1, that is dose-dependent, reinforces the correct interpretation of the negative association between downregulation of the *MYCL, MYCN* and *ASCL1* genes and sensitivity to JQ1, because it serves as an internal comparative control. To define the critical and direct targets of JQ1 for growth suppression of SCLC cells, the effect of JQ1 on transcriptional regulation of those genes should be further examined by several other methods, such as a chromatin immunoprecipitation assay.

In this context, JQ1 sensitivities in various SCLC cell lines have been also shown recently by two other studies [20, 35], with several cell lines overlapping among the three studies. However, the GI_50_ values of several cell lines were considerably different in these studies (Supplementary Table S2), likely a consequence of the difficulty to perform drug screening using SCLC cells in culture, as described in Materials and Methods. Thus, development of proper drug screening methods will be very important for the identification of novel drugs for the treatment of SCLC patients. Recently, several BET bromodomain inhibitors other than JQ1 has been used in clinical trials in solid tumors [38]. Therefore, it is also very important to assess the similarity and difference in the effect of those inhibitors. Since several CDK4/CDK6 inhibitors have been also used in clinical trials in various types of cancers [39], it would be worth investigating the effectiveness of combinational therapies using BET bromodomain inhibitors as well as CDK6 inhibitors for SCLC patients.

The fusion of the *MYCL* gene with the *RLF* gene is likely to have a pathogenic role in SCLC progression, because this fusion has been recurrently detected among several studies [6, 8, 40]. All the *RLF-MYCL* fusion genes detected up to now express the same fusion protein of 446 amino acids encoded by exon 1 of *RLF* in frame with exons 2 and 3 of *MYCL* leading to the expression of a fusion protein composed of the first 79 amino acids of RLF and a MYCL protein lacking its first 27 amino acids. Therefore, this fusion gene is likely to be transcribed by using the *RLF* gene promoter. In this line, JQ1 did not reduce the amount of RLF/MYCL fusion protein, while it reduced the amount of wild-type MYCL protein in the H1963 cell line. Consistently, *MYCL* mRNA was not reduced in H1963 but reduced in three other *MYCL* amplified cell lines. Therefore, BRD2, BRD3 and/or BRD4, critical functional targets of JQ1, seems to bind to the promoter region of *MYCL* but not to that of *RLF*.

In conclusion, our results show the promising use of MYCL as a target for therapy for SCLC by the use of epigenetic inhibitors of its expression. It is known that *MYCL* is dispensable for embryonic development and *MYCL* expression is highly restricted with respect to tissue and developmental stage [10-12]; therefore, MYCL inhibition would predictably not have a severe side effect in non-cancerous tissues. Although MYCL is only amplified in around 10% of SCLCs, our data shows it is expressed in the majority of the SCLC cell lines widening the potential impact for therapy.

## MATERIALS AND METHODS

### Cell lines

Fourteen SCLC cell lines, HCC33, H2141, H1963, H1184, H209, H345, H1618, H2107, Lu135, H82, H69, H526, Lu134 and Lu165, were used in this study. HCC33, H1963, H345, H1618, H2107, H82 and H69 were obtained from Dr. J. D. Minna (University of Texas Southwestern, Dallas, USA), H526 from Dr. C. C. Harris (National Institute of Health, Bethesda, USA), Lu135 and Lu134 from Dr. T. Terasaki (National Cancer Center, Tokyo, Japan), Lu165 from RIKEN BioResource Center (Ibaraki, Japan), and H2141, H1184 and H209 from the American Type Culture Collection (Manassas, USA). Cells were cultured in RPMI medium supplemented with 10% FBS with 5% CO_2_ at 37°C.

### Cell growth assay

The population doubling times of SCLC cell lines are generally very long, and those in the 14 cell lines used in this study ranged from 3 to 7 days. The cells proliferate as multi-cell floating aggregates in *in vitro* cultures. These properties make the use of SCLC cell lines difficult for drug screening due to the following reasons. First, if cell growth is assessed in 2 to 3 days after drug addition, as in other cancer cell lines, the number of non-treated SCLC cells often does not double at the time of assessment, compared to the number of plated cells. Therefore, if a drug has an effect to induce only growth arrest but not cell death, the GI_50_ (50% growth inhibition) values would be very high [18]. For this reason, the cells were cultured for 3 to 7 days depending on the doubling time of each cell line. The concentration of the cells at the time of drug addition was defined individually among the cell lines to obtain the exponential growth of the cells. Second, if the aggregated cells are treated, it is difficult to obtain accurate cell numbers at the time of plating and assessment. Third, due to the aggregation of cells, it is possible that the cells are not evenly exposed to the drugs in culture. Therefore, the cells were treated with trypsin at the time of drug addition and effect evaluation, and cell number and viability were determined by a standard dye exclusion trypan blue assay. For each sample, at least three counts were performed and standard deviation was calculated. Serially diluted JQ1 (#4499, Tocris Bioscience) dissolved in DMSO were added to the medium to obtain a concentration of 0.1-10.0 μM of JQ1 in 0.1% DMSO. Previously, the effect of JQ1 on the growth of SCLC cell lines was assessed 3 days and 4 days after JQ1 addition by the MTS dye conversion method (Promega) and with ATP content (CellTiter Glo®, Promega), respectively [20, 37].

### Western blot analysis

Lysate preparation and Western blot analyses were performed as described previously [34]. Antibodies used are MYCL (AF4050, R&D), MYC (sc-40, Santa Cruz), MYCN (#9405, Cell Signaling), MAX (sc-197, Santa Cruz), ASCL1 (556604, BD Pharmingen), p21 (#2947, Cell Signaling), PARP-1 (#9542, Cell Signaling), and α-Tubulin (CP06, CalBiochemicals).

### mRNA microarray analysis

Total RNA was extracted using Maxwell® 16 LEV simplyRNA Tissue Kit (Promega) and qualified with a model 2100 Bioanalyzer (Agilent). All samples showed RNA Integrity Numbers higher than 7.9 and were subjected to microarray experiments. Ten nanograms of total RNA were processed and hybridized to GeneChip® Human Transcriptome Array 2.0 (Affymetrix, Santa Clara, CA, USA) using the GeneChip® WT Pico Reagent Kit (P/N 703262 Rev. 2). After hybridization, arrays were washed and stained in the Affymetrix GeneChip Fluidics Station 450 and scanned in the Affymetrix GeneChip Scanner 3000 7G. The data were processed by the MAS5 algorithm, and the mean expression level of a total of 67,528 genes was adjusted to 1,000 for each sample.

## SUPPLEMENTARY MATERIALS FIGURES AND TABLES


