# Supplementary material for: MYCL is a target of a BET bromodomain inhibitor, JQ1, on growth suppression efficacy in small cell lung cancer cells
Source: Oncotarget. 2016 Oct 14;7(47):77378–88. doi: 10.18632/oncotarget.12671 (PMC5363592; doi:10.18632/oncotarget.12671)
Supplement: Supplementary file 1 [file oncotarget-07-77378-s001.pdf]

MYCL is a target of a BET bromodomain inhibitor, JQ1, on growth suppression efficacy in small cell lung cancer cells

SUPPLEMENTARY FIGURES AND TABLES

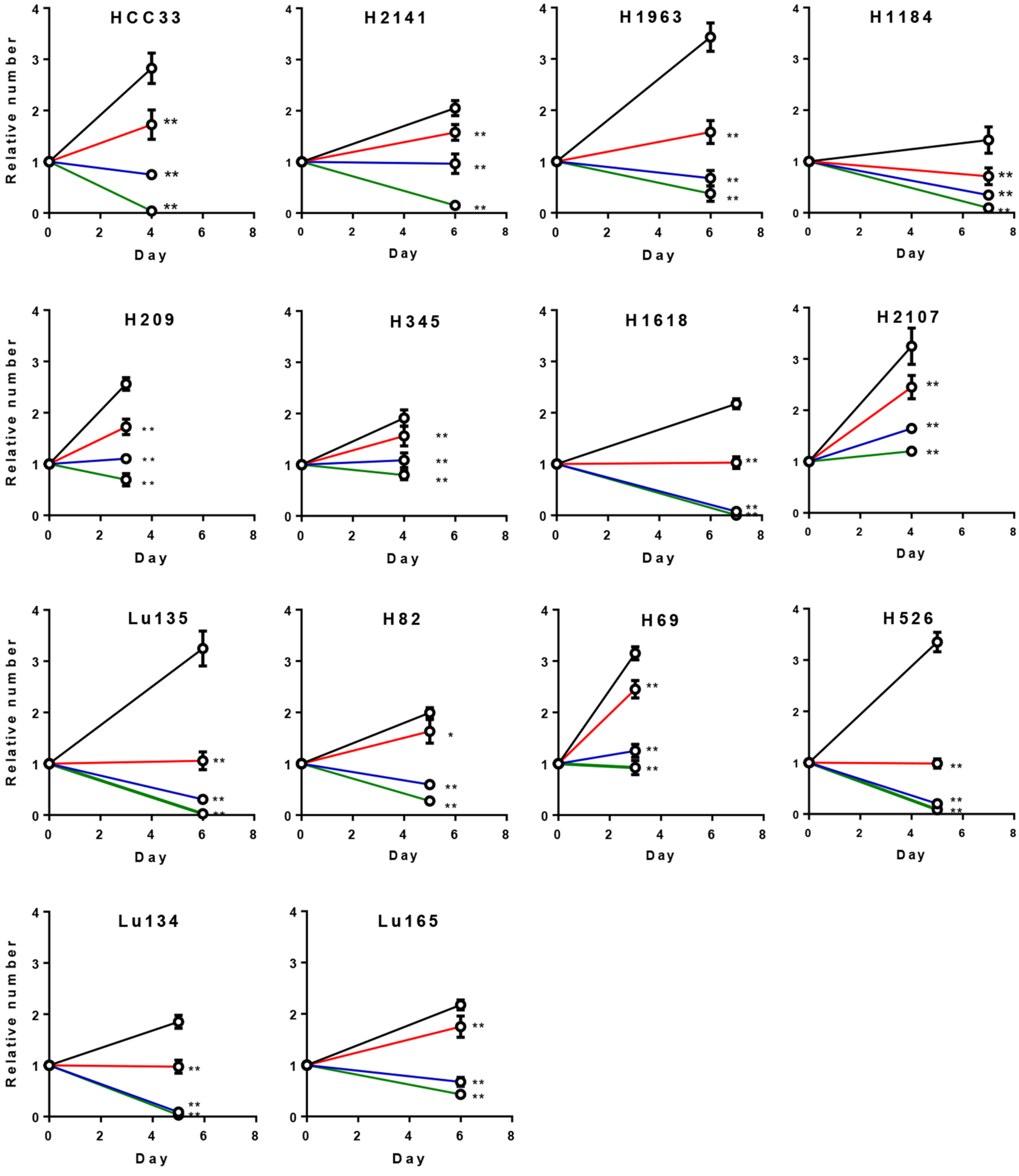

Supplementary Figure S1: Growth reduction of SCLC cell lines by JQ1 treatment. The cells were cultured for 3 to 7 days with and without JQ1. Black, red, blue, and green lines represent 0.0, 0.1, 1.0, and 10.0 µM of JQ1-treated cells, respectively. The numbers of cells were counted by a dye exclusion trypan blue assay at the time of plating (day 0) and after 3 to 7 days of JQ1 treatment. *p<0.05, **p<0.01 by unpaired t-test.

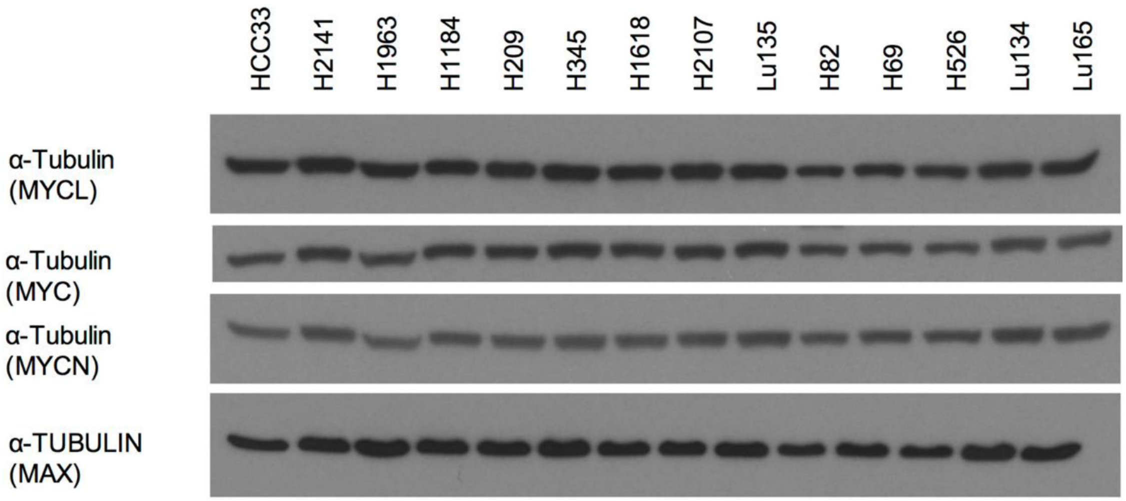

Supplementary Figure S2: Expression of α-Tubulin in 14 SCLC cell lines. These four membranes were used for detection of MYCL, MYC, MYCN and MAX proteins, respectively, in Figure 1.

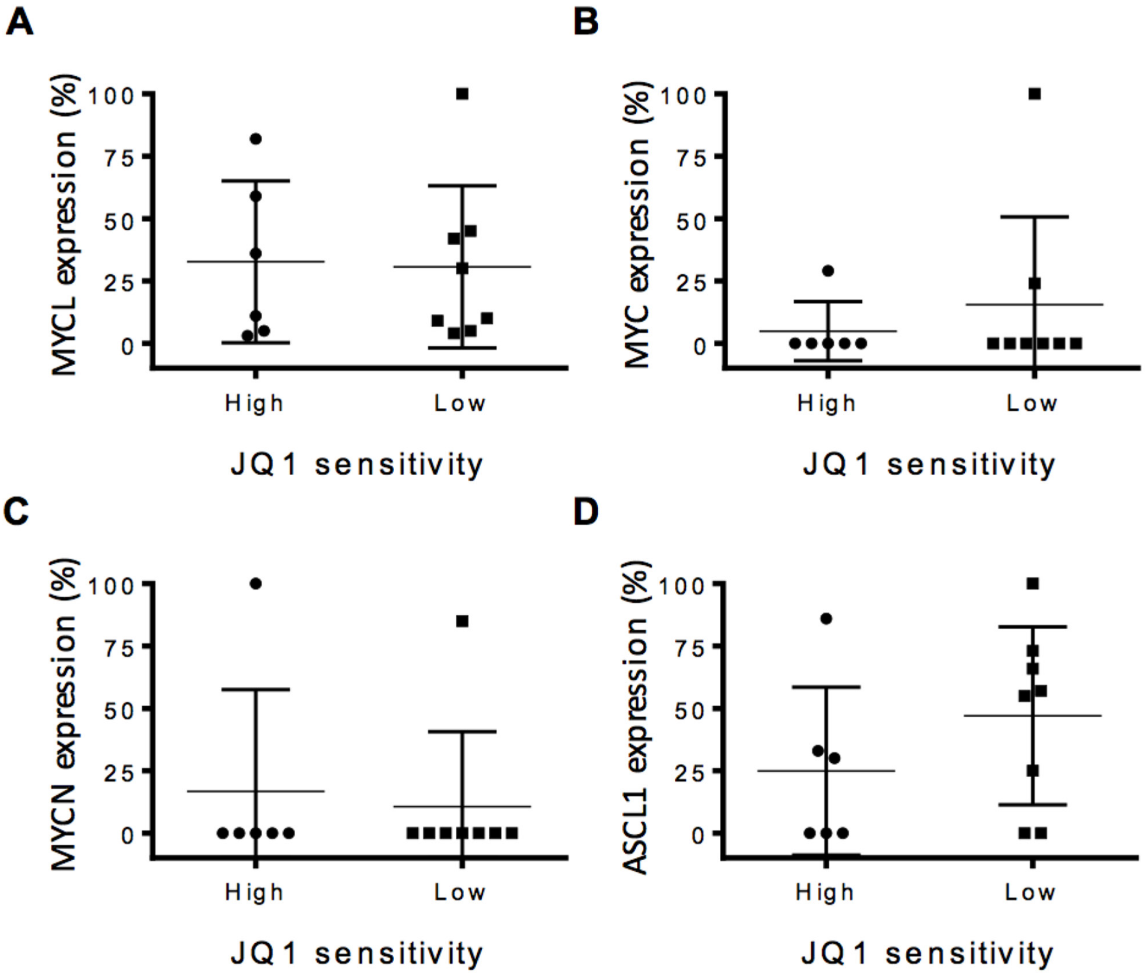

Supplementary Figure S3: Association between JQ1 sensitivities and expression of MYCL, MYC, MYCN and ASCL1 proteins in SCLC cell lines. Cell lines with GI_{50} less than 1 μM and more than 1μM were defined as high and low sensitive lines to JQ1. There were no significant associations between JQ1 sensitivity and expression of MYCL **A.** MYC **B.** MYCN **C.** and ASCL1 **D.**

Supplementary Table S1: Effects of JQ1 on the transcriprion of *MYCL* and known JQ1 target genes in SCLC cell lines

Cell line	JQ1 treatment	MYCL	MYCN	MYC	ASCL1	FOSL1	BCL2	CDK6	IL7R	RUNX2	CDC25B
HCC33	-	9570	407	243	353	426	675	442	64	192	2350
	+	8230	391	265	381	443	624	315	68	201	2254
H2141	-	3827	463	245	7708	434	997	272	72	188	2954
	+	2722	437	247	6747	457	768	286	69	200	2531
H1963	-	6833	361	216	2655	411	561	499	65	190	1888
	+	7354	412	241	1006	429	458	485	65	216	1490
H345	-	1144	445	273	8560	467	2856	297	72	201	2422
	+	1016	595	339	7164	583	1556	349	79	258	1877
H1618	-	2873	566	253	8200	484	998	1227	70	263	3126
	+	1947	492	238	8551	446	707	897	67	231	2308
H2107	-	981	425	1559	8480	513	2223	298	76	202	2448
	+	921	448	1966	4682	603	1293	304	79	214	2233
Lu135	-	587	364	1923	354	406	452	1501	70	177	2630
	+	619	345	2207	224	416	443	668	67	192	2051
H82	-	996	456	4829	509	354	348	1354	64	209	1373
	+	1048	433	4929	323	363	317	877	61	194	1082
H69	-	669	1004	267	4865	451	915	417	68	192	2302
	+	614	821	267	2689	408	607	334	69	177	2504
H526	-	776	1662	226	234	428	995	1229	65	280	2746
	+	759	1333	247	305	496	619	709	68	261	2587
Lu134	-	661	372	258	2533	433	491	1095	68	193	2847
	+	618	366	278	2265	420	350	587	67	184	2687
Lu165	-	775	418	247	7987	420	501	389	68	203	2068
	+	839	415	241	4114	417	348	340	64	182	1583

Supplementary Table S2: Comparison of GI_{50} values for JQ1 in SCLC cell lines among three studies

	GI_{50} value (µg/ml)		
**Cell line**	**This study**	**Lenhart et al.**	**Kaur et al.**
Lu134	<0.1		
H1184	<0.1		
H526	<0.1	0.994	0.11
H1618	<0.1		
Lu135	<0.1	0.015	
H1963	<0.1		0.26
HCC33	0.13	0.063	
H82	0.18	1.140	1.00
Lu165	0.19		
H2141	0.22	0.989	
H209	0.52		
H69	0.54	0.144	0.23
H2107	1.1		0.13
H345	1.23		>10.0
H211		0.025	0.23
H446		0.035	0.12
H2171		0.175	0.22
H196		4.27	1.16

GI_{50} values of these cell lines were obtained from the data shown in Figure 2 in this study and from references 20 and 35. The GI_{50} values were not described in the paper by Kaur et al., thus, were calculated from the data shown in Figure 4 in the paper.
